# Radiosensitizing capacity of fenofibrate in glioblastoma cells depends on lipid metabolism

**DOI:** 10.1016/j.redox.2024.103452

**Published:** 2024-12-02

**Authors:** Bayan Alkotub, Lisa Bauer, Ali Bashiri Dezfouli, Khouloud Hachani, Vasilis Ntziachristos, Gabriele Multhoff, Morteza Hasanzadeh Kafshgari

**Affiliations:** a Institute of Biological and Medical Imaging (IBMI), Helmholtz Zentrum München, Neuherberg, Germany; b Chair of Biological Imaging at the Central Institute for Translational Cancer Research (TranslaTUM), TUM School of Medicine and Health, Klinikum rechts der Isar, Technical University of Munich, Munich, Germany; c Radiation Immuno-Oncology Group, Central Institute for Translational Cancer Research (TranslaTUM), TUM School of Medicine and Health, Klinikum rechts der Isar, Technical University of Munich (TUM), Munich, Germany; d Department of Radiation Oncology, TUM School of Medicine and Health, Klinikum rechts der Isar, Technical University of Munich (TUM), Munich, Germany; e Department of Otorhinolaryngology, TUM School of Medicine and Health, Klinikum rechts der Isar, Technical University of Munich (TUM), Munich, Germany

**Keywords:** Fenofibrate, Glioblastoma, Radiotherapy, Lipid droplets, DGAT1, GPAT4, Biomimetic lipid nanoparticles

## Abstract

Despite advances in multimodal therapy approaches such as resection, chemotherapy and radiotherapy, the overall survival of patients with grade 4 glioblastoma (GBM) remains extremely poor (average survival time <2 years). Altered lipid metabolism, which increases fatty acid synthesis and thereby contributes to radioresistance in GBM, is a hallmark of cancer. Therefore, we explored the radiosensitizing effect of the clinically approved, lipid-lowering drug fenofibrate (FF) in different GBM cell lines (U87, LN18). Interestingly, FF (50 μM) significantly radiosensitizes U87 cells by inducing DNA double-strand breaks through oxidative stress and impairing mitochondrial membrane integrity, but radioprotects LN18 cells by reducing the production of reactive oxygen species (ROS) and stabilizing the mitochondrial membrane potential. A comparative protein and lipid analysis revealed striking differences in the two GBM cell lines: LN18 cells exhibited a significantly higher membrane expression density of the fatty acid (FA) cluster protein transporter CD36 than U87 cells, a higher expression of glycerol-3-phosphate acyltransferase 4 (GPAT4) which supports the production of large lipid droplets (LDs), and a lower expression of diacylglycerol O-acyltransferase 1 (DGAT1) which regulates the formation of small LDs. Consequently, large LDs are predominantly found in LN18 cells, whereas small LDs are found in U87 cells. After a combined treatment of FF and irradiation, the number of large LDs significantly increased in radioresistant LN18 cells, whereas the number of small LDs decreased in radiosensitive U87 cells. The radioprotective effect of FF in LN18 cells could be associated with the presence of large LDs, which act as a sink for the lipophilic drug FF. To prevent uptake of FF by large LDs and to ameliorate its function as a radiosensitizer, FF was encapsulated in biomimetic cell membrane extracellular lipid vesicles (CmEVs) which alter the intracellular trafficking of the drug. In contrast to the free drug, CmEV-encapsulated FF was predominantly enriched in the lysosomal compartment, causing necrosis by impairing lysosomal membrane integrity. Since the stability of plasma and lysosomal membranes is maintained by the presence of the stress-inducible heat shock protein 70 (Hsp70) which has a strong affinity to tumor-specific glycosphingolipids, necrosis occurs predominantly in LN18 cells having a lower membrane Hsp70 expression density than U87 cells.

In summary, our findings indicate that the lipid metabolism of tumor cells can affect the radiosensitizing capacity of FF when encountered either as a free drug or as a drug loaded in biomimetic lipid vesicles.

## Highlights

• The radiosensitizing effect of fenofibrate in glioblastoma cells depends on lipid metabolism.
• Fenofibrate enhances radiosensitivity in glioblastoma cells with small LDs but protects glioblastoma cells with large LDs.
• Radioprotective effects of large LDs can be reversed by altering drug delivery and intracellular drug trafficking.

## Graphical abstract

**Figure:**
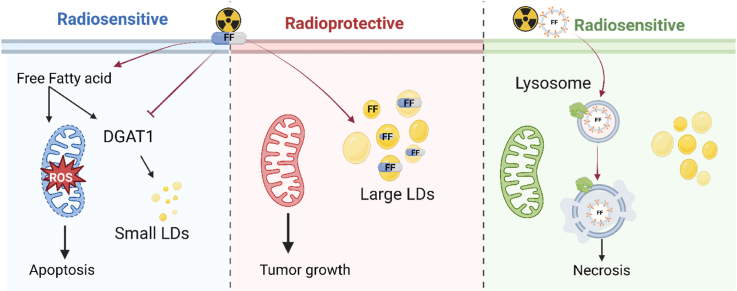

## 1 Introduction

Glioblastoma (GBM) is the most prevalent type of primary brain tumor and is associated with an exceedingly poor prognosis and diminished quality of life [1,2]. Although advances in therapeutic regimens have improved outcomes, the median overall survival of GBM remains extremely poor (10–20 months) owing to its high potential for recurrence and development of therapy resistance [3,4]. Radiotherapy (RTx) concomitant with the alkylating agent temozolomide (TMZ) is one of the main pillars of GBM therapy following surgical tumor resection [5]. RTx induces DNA damage either directly or indirectly via the production of reactive oxygen species (ROS) [6]. Unlike systemic chemotherapy, RTx is delivered locally to the tumor, however the surrounding normal tissue limits its dose [7]. Moreover, radioresistance is a significant challenge, as tumor cells adapt to radiation-induced changes and acquire resistance to radiation-induced damage. Radioresistance is mainly led by DNA damage repair, cell cycle arrest, changes in the tumor microenvironment (TME), the generation of cancer stem cells, and alterations in tumor cell metabolism (e.g., Warburg effect and lipid metabolism) [[8], [9], [10], [11], [12]].

Interest in lipid metabolism as a hallmark of cancer has grown since an increased processing of fatty acids (FAs) was found to be involved in mediating radioresistance in nasopharyngeal carcinoma, breast cancer, and thyroid cancer [[13], [14], [15]]. FAs produced by *de novo* fatty acid synthesis via fatty acid synthase (FASN) or by the exogenous uptake of lipids from the TME (e.g., through the scavenger receptor CD36 [16,17]) fuel rapid tumor cell growth [18]. Despite the vital role of FAs in different cellular processes, an excess of FAs harms cells and causes lipotoxicity [19]. Therefore, excess free FAs get packaged into lipid droplets (LDs) on the endoplasmic reticulum (ER) surface after conversion into triacylglycerol (TAG), a process which is facilitated by various lipid synthase enzymes including diacylglycerol O-acyltransferase (DGATs) and glycerol-3-phosphate acyltransferases (GPATs) [20]. Beyond their storage function, LDs also act as cytoplasmic chaperones during cellular stresses (e.g., ER, oxidative and nutrient stress), mitochondrial dysfunction, and energetic and redox imbalances [21,22]. Furthermore, LDs contribute to RTx failure by attenuating the radiation-induced cellular stress response [23].

Anti-hypercholesterolemia and -hyperlipidemia drugs such as statins and fibrates can trigger apoptosis in GBM [24,25]. Moreover, the FDA-approved drug fenofibrate (FF) has a lipid-lowering effect by activating the peroxisome proliferator-activated receptor α (PPAR α) [26]. Although FF has been shown to exert some anti-cancer activities, its effect in combination with RTx treatment on the radiation responses of GBM (e.g., radiosensitizing through lipid metabolism modulation) has not yet been explored.

As shown in earlier studies, a high lipid metabolism in GBM cells can trigger resistance to lipophilic drugs such as TMZ and suppress their therapeutic efficacy [27]. To ameliorate their efficacy, lipophilic drugs such as TMZ or FF can be encapsulated in biomimetic nanocarriers (e.g., exosomes and extracellular microvesicles) to alter their uptake route and intercellular pathways and to break resistance mechanisms induced by an altered lipid metabolism. In addition, such nanocarriers can reduce systemic side effects of the administered free drug and overcome biological barriers such as the blood-brain barrier [[28], [29], [30]]. Cancer membrane-derived extracellular vesicles (CmEVs), having a wide range of functional cell membrane receptors and proteins, have been implemented to extend the circulation time of drugs and enable homologous targeted accumulation and drug release in the TME with minimal side effects compared to synthesized organic and inorganic nanocarriers [[31], [32], [33], [34]]. Using CmEVs as carriers for FF is attractive because their biomimetic features derived from biological cell membranes can minimize the risk of inflammation and homologous interactions and facilitate integration with RTx, leading to highly efficient cancer therapy.

To develop a more efficient RTx approach for treating GBM tumors, this study explores the potential of FF as a radiosensitizing agent, both as a free drug and encapsulated in biomimetic lipid nanoparticles (CmEVs). Furthermore, we examined the mechanism of action of FF in combination with RTx in GBM cell lines that differ in their lipid metabolism. We identify the presence of large LDs as a mediator of radiation resistance in LN18 tumor cells, which could be overcome by altering the delivery system of FF.

## 2 Results

### 2.1 FF mediates opposite effects on radiosensitivity in two GBM cell lines

**Fig. 1:**
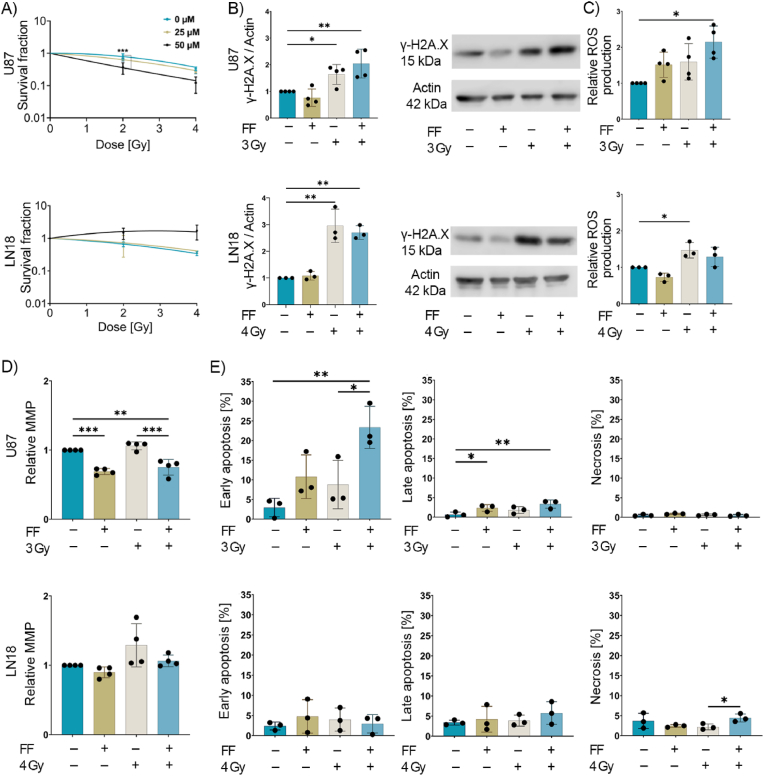
**Effect of fenofibrate (FF) treatment on the radiosensitivity and apoptosis of glioblastoma (GBM) cell lines.** A) Clonogenic cell survival assay of untreated (0 μM) and FF-treated (25 or 50 μM) U87 and LN18 cells after irradiation (RTx) with 0 Gy (sham), 2 Gy, and 4 Gy. The surviving fraction was normalized to the surviving fraction in the sham group, which was used as a control. The values plotted represent the mean of 3 independent experiments performed in duplicate (n = 3; ± SD). B) Western blot analysis of γH2AX in U87 (top) and LN18 (bottom) cells 30 min post-RTx (24 h after 25 μM FF treatment). Actin was used as an internal control, and all intensities were normalized to the control group. C) Graphs showing the impact of FF and combined (FF + RTx) treatment on reactive oxygen species (ROS) production in U87 (top) and LN18 (bottom) cells as measured by the DCFDA probe. D) The relative mitochondrial membrane potential (MMP) in U87 (top) and LN18 (bottom) cells after treatment. E) The percentage of early and late apoptotic cells and necrotic cells are plotted for all treatments and for both U87 (top row) and LN18 (bottom row) cells. In all bar graphs, data are plotted as means ± SD of at least 3 independent experiments. P value was calculated using a two-way analysis of variance (ANOVA) with Tukey's correction. ∗p < 0.0332, ∗∗p < 0.0021, ∗∗∗p < 0.0002.

Human GBM cell lines (U87 and LN18) were treated with increasing concentrations of FF (0, 12.5, 25, 50, and 100 μM) for 24 h. Cell viability was then determined using a CellTiter-Glo® luminescent cell viability assay which measures adenosine triphosphate (ATP) production. At concentrations lower than 25 μM, FF did not cause any significant toxicity in either GBM cell line (Supplementary Fig. S1). The impact of FF on the radiation response of U87 and LN18 cells was evaluated in colony formation assays. For this, cells were treated with FF (0 (control), 25, 50 μM) for 24 h followed by an exposure to a single radiation dose of 0 (sham), 2 and 4 Gy. Treating U87 cells with FF (25 μM) in combination with a sub-lethal irradiation dose of 2 Gy resulted in a decline in the survival of U87 cells which reached significance at an FF concentration of 50 μM (Fig. 1A). In contrast, the survival of LN18 cells was not affected after a combined treatment with FF (25 μM) and 2 Gy, and at a concentration of 50 μM FF appears to mediate a radioprotective effect (Fig. 1A). As summarized in Table 1, the radiation dose required to inactivate 50 % of U87 cells (D_50_) declined from 3.64 to 2.92 Gy in combination with 25 μM FF and to 1.73 Gy in combination with 50 μM FF. However, in LN18 cells D_50_ increased from 2.95 to 3.39 at an FF concentration of 25 μM and to 7.26 Gy at an FF concentration of 50 μM.

**Table 1:** Radiobiological parameters after exposure to fenofibrate (FF).

Cell Line	FF [μM]	D_50_ [Gy]^a^	SER^b^	α [Gy^-1^]^c^	β [Gy^-1^]^c^
U87	0	3.64	1.00	−0.14	0.09
	25	2.92	1.25	0.08	0.05
	50	1.73	2.11	0.41	0.00
LN18	0	2.95	1.00	0.14	0.03
	25	3.39	0.87	0.10	0.03
	50	7.26	0.41	−0.37	0.06

a D_50_, dose [Gy] required for 50 % inactivation of a tumor cell population.

b SER, Sensitizing enhancement ratio = D_50_ (control)/D_50_ (drug treatment). A SER greater than 1.20 indicates radio sensitization.

c α and β values were derived from the linear quadratic equation f = exp (-α ∗x- β ∗x2).

Based on the radiobiological parameters after exposure to FF shown in Table 1, we pre-treated U87 and LN18 cells with the sub-lethal dose of 25 μM FF for 24 h and exposed them to a single radiation dose of 3 Gy (U87) and 4 Gy (LN18) to kill approximately 50 % of the tumor cell population. After exposure of U87 and LN18 cells to these radiation doses in the absence of FF, the formation of γH2AX protein complexes was examined as a measure of DNA double-strand breaks (DSBs) which is indicative of radiosensitivity [6]. An irradiation of U87 and LN18 cells with 3 Gy and 4 Gy, respectively caused a significant increase in γH2AX protein complexes in both cell lines (Fig.1B) which was further increased in U87 cells when cells were pre-treatment with FF (25 μM, 24 h). In contrast, a combined treatment of FF (25 μM, 24 h) and irradiation (4 Gy) did not further enhance the radiosensitivity of LN18 cells. A combined treatment of FF and RTx resulted in a mild reduction in γH2AX protein complexes when compared to the RTx group (Fig.1B). These findings provide a first hint that pre-treatment with FF before irradiation might radiosensitize U87 cells, but radioprotect LN18 cells.

Ionizing radiation is known to generate reactive oxygen species (ROS) that impair the mitochondrial membrane potential (MMP) [6], induce DNA damage and lead to apoptotic cell death [35]. DCFDA (2′,7′-Dichlorofluorescin diacetate), which detects hydrogen peroxide, hydroxyl radicals, carbonate radicals and nitrogen dioxide and Dihydroethidium (DHE) which detects cytosolic superoxide, hydroxyl radicals and peroxynitrite were used to detect different ROS species following treatment of U87 and LN18 with FF, RTx and a combination of FF and RTx [36]. In U87 cells, a pre-treatment with FF or RTx (3 Gy) resulted in a moderate upregulation of ROS which reached statistical significance after a combined treatment with FF and RTx (3 Gy) (Fig. 1C, Supplementary Fig. S2), whereas radiation-induced increased ROS levels in LN18 were not further increased by a combined treatment of FF followed by RTx (4 Gy) (Fig. 1C, Supplementary Fig. S2). In addition to ROS, the MMP was assessed in both GBM cell lines by staining with Tetramethylrhodamine-ethyl ester (TMRE). In U87 cells, but not in LN18 cells, a significant reduction in MMP to below control levels was induced by treatment with FF alone or after a combined treatment with FF and RTx (3 Gy) (Fig. 1D).

To determine the mechanism of cell death after a singular or combined treatment with FF and RTx, ferroptosis was measured using the ferroptosis marker GPx4 [37] in both cell types upon treatment. As shown in Supplementary Fig. S3, expression of the ferroptosis marker GPx4 did not differ significantly in either cell type upon treatment with FF, RTx or a combined treatment. Next, we tested whether FF induces apoptosis using the Annexin V/Propidium Iodide (PI) apoptosis assay. A combined treatment with FF (25 μM) and RTx (3 Gy) significantly increased early apoptosis in U87 cells which was significantly higher than that of RTx alone, although an irradiation with the sublethal dose of 3 Gy without FF did not induce a significant change in apoptosis in U87 cells. A significant increase in late apoptotic cells at low percentages was observed after a treatment with FF (25 μM) and a combined treatment with FF and RTx in U87 cells (Fig. 1E). Necrosis was not induced in U87 cells after any treatment. In LN18 cells no significant increase in early and late apoptosis was observed, irrespective of the treatment regimen with FF and RTx (4 Gy) (Fig. 1E). Taken together, these findings demonstrate that the administration of FF induces differential radiation responses with respect to ROS, MMP and apoptosis in U87 and LN18 cells.

### 2.2 Radioprotective effects of FF in GBM cells are mediated by large lipid droplets

**Fig. 2:**
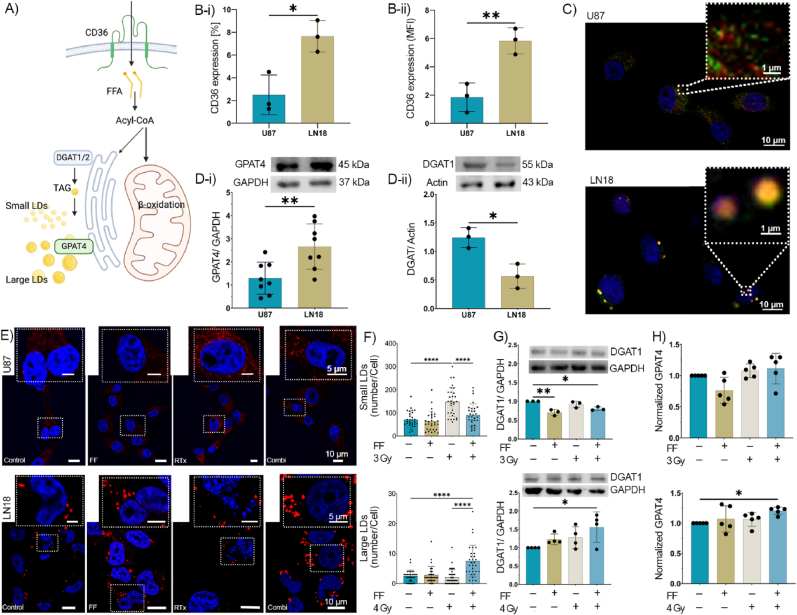
**Fenofibrate (FF) modulates lipid droplets (LDs) in U87 and LN18 cells.** A) Schematic representation of uptake and processing pathways of fatty acids (FAs) in tumor cells. B-i) Percentage of U87 and LN18 cells expressing CD36. B-ii) Mean fluorescent intensity (MFI) of CD36 expression in U87 and LN18 cells. C) U87 and LN18 cells were stained with LipidTOX™ (red) for LD, GPAT4 antibody (green) for lipid enzyme and Hoechst (blue) for nuclei. D) Representative immunoblots and bar graphs (from 3 independent experiments) of basal GPAT4 (D-i) and DGAT1 (D-ii) expression in U87 and LN18 cells. E) Representative confocal images of LDs (red) and nuclei (blue) of U87 and LN18 cells for all treatment groups. F) Graph showing numbers of small and large LDs in U87 (top) and LN18 (bottom) cells per treatment. LD quantification was performed by counting red lipid bodies using ImageJ (minimum of 50 cells per cell line). G) Representative immunoblots and bar graphs showing the expression of DGAT1 in U87 (top) and LN18 (bottom) cells after FF and RTx treatment. H) The normalized value of intracellular GPAT4 in U87 (top) and LN18 (bottom) cells in all treatment groups. Unless stated otherwise, data are shown as means ± SD of at least three independent experiments. P values were calculated using an unpaired *t*-test (two groups) or a two-way analysis of variance (ANOVA, more than two groups) with Tukey's correction. ∗p < 0.0332, ∗∗p < 0.0021, ∗∗∗p < 0.0002 and ∗∗∗∗p < 0.0001.

As lipid metabolism is known to play an important role in tumor progression and drug resistance [27,38] we asked whether the differential effects of FF on the radiosensitivity of U87 and LN18 cells might be explained by differences in the lipid metabolism, such as differences in the uptake of lipids from the extracellular milieu via the scavenger receptor CD36. CD36 is critical for FA uptake from the microenvironment into the cytosol and facilitates energy-dependent FA metabolism through mitochondria-mediated β-oxidation (Fig. 2A) [39]. Flow cytometry was used to determine the expression of the FA transporter CD36 [17] on the cell membrane of U87 and LN18 cells in order to elucidate potential differences in the exogenous uptake of FA in GBM cells. The expression density and proportion of positively stained cells of CD36 was significantly higher in LN18 cells than in U87 cells (Fig. 2B-i/ii) and a treatment with FF, RTx and a combination of both did not significantly alter the expression of CD36 in either cell line (Supplementary Fig. S4).

To maintain lipid homeostasis and prevent lipotoxicity, cancer cells store converted excessive FA in small and large LDs whose production depends on the enzymes DGAT1 and GPAT4, respectively (Fig. 2A). The total LD content and the number and size of LDs before and after treatment with FF and irradiation were evaluated by intracellular neutral lipid staining using HCS LipidTOX™ Red Neutral Lipid Stain. Under physiological conditions (control), U87 cells contained high numbers of small LDs; LN18 cells had fewer but very large LDs (LD size ∼20 times larger compared to those in U87 cells) (Fig. 2C and E). A co-immunostaining showed that GPAT4 (green), an ER-residing enzyme, is predominantly enriched in large LDs (red) of LN18 cells (Fig. 2C). The higher expression of GPAT4 in LN18 cells compared to that of U87 cells (Fig. 2D-i) promotes the formation of large LDs and the enlargement of LDs after translocation to the LD surface [40]. In addition, the greater expression of DGAT1 in U87 cells plays a role in forming small LDs compared to the lower expression of LN18 cells (Fig 2D-ii). Treatment with FF alone did not significantly affect the amount of small and large LDs in U87 and LN18 cells (Fig. 2E and F). Upon irradiation (3 Gy), the number of small LDs increased in U87 cells but dropped to basal levels when cells were pre-treated with FF before irradiation. Although radiation did not affect the number of large LDs in LN18 cells, a combined treatment with FF and RTx (4 Gy) resulted in a significant up-regulation of large LDs in these cells (Fig. 2E and F). With respect to DGAT1, an enzyme that promotes the production of LDs to avoid lipotoxicity [40], a dual treatment with FF and RTx resulted in an increased expression in LN18 cells and a decreased expression in U87 cells (Fig. 2G) that initially contained higher DGAT1 levels than LN18 cells (Fig. 2D-ii). The increase in the number of large LDs in LN18 cells after combined treatment (Fig. 2E and F) is likely associated with a significantly elevated expression of GPAT4 (Fig. 2H), whereas in U87 cells containing small LDs (Fig. 2C and E), GPAT4 remained unaltered upon any treatment (Fig. 2H). These results might explain the heterogeneous responses of U87 and LN18 to treatment with FF and RTx.

Perilipin 5 (PLIN5) inhibits the activity of adipose triglyceride lipase (ATGL) which is responsible for the lipolysis/degradation of LDs. To investigate the stability of small and large LDs in U87 and LN18 cells, we determined the expression of PLIN5 before and after the different treatments. Since neither FF, RTx or a combined treatment impaired PLIN5 expression in U87 and LN18 cells, we assume that lipolysis or degradation of LD are not responsible for the differential effects of FF on the radiosensitivity of U87 and LN18 cells (Supplementary Fig. S5) [41,42]. Nevertheless, combining FF and RTx significantly increased DGAT1 expression in LN18 cells and decreased DGAT1 expression in U87 cells. Therefore, we assume that the FF-induced reduction in the LD content of U87 cells is mediated via a reduction in DGAT1 (Fig. 2G). Intracellular levels of GPAT4 remained low and unaltered in U87 cells across all treatment groups but increased significantly in LN18 cells after a combined treatment with FF and RTx (Fig. 2H).

**Fig. 3:**
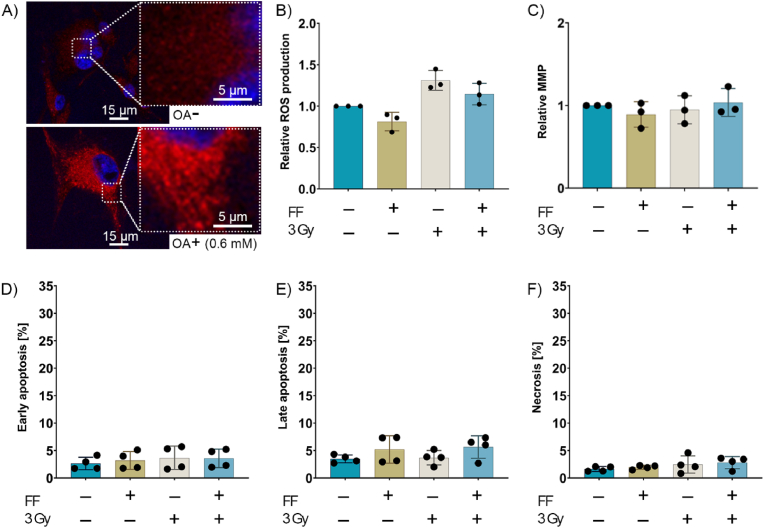
**Effect of fenofibrate on U87 cells containing large lipid droplets (LDs).** A) Confocal images of U87 cells untreated and after treatment with oleic acid (OA; 600 μM); small and large LDs (red), nuclei (blue). Effects of FF, irradiation (RTx) and a combi-treatment (FF + RTx) on the ROS production (B), mitochondrial membrane potential (MMP) (C), early apoptosis (D), late apoptosis (E) and necrosis (F) of U87 cells containing large LDs after treatment with OA. Data represent mean values ± SD of three to four independent experiments.

To validate the radioprotective effect of large LDs within an autologous tumor cell system, U87 cells were treated with oleic acid (OA, 600 μM) which stimulates the production of large LDs [40]. The existence of large LDs in U87 cells after treatment with OA was confirmed by confocal microscopy (Fig. 3A). A treatment of U87 cells containing large LDs with FF (25 μM) alone or in combination with RTx (3 Gy) did not induce ROS production (Fig. 3B) and did not alter the MMP (Fig. 3C). As a consequence, early/late apoptosis or necrosis was not induced in U87 cells containing large LDs (Fig. 3D-F). These findings strengthen the hypothesis that large LDs mediate the radioprotective effects upon FF treatment.

Due to their hydrophobic surfaces, large LDs in LN18 cells could absorb most of the internalized hydrophobic free drug FF and thereby suppress the radiosensitizing effects of FF in the mitochondrial compartment [43,44]. The obvious link between LD size and FF resistance makes it necessary to develop new strategies to block the uptake of FF into large LDs. Inhibition of key enzymes such as cPLAα, which is involved in the formation of large LDs [45], or changing the formulation of FF to alter the intracellular trafficking of the drug might be able to reduce radioresistance in LN18 cells.

### 2.3 FF-loaded lipid nanoparticles break the radioresistance of GBM cells

**Fig. 4:**
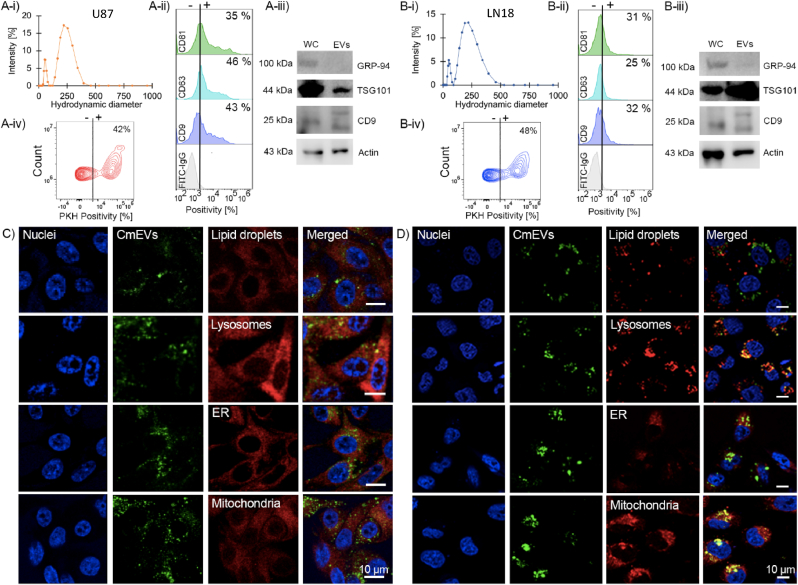
**Characterization and intracellular trafficking of cancer cell membrane-derived extracellular vesicles (CmEVs) loaded with fenofibrate (FF) in U87 and LN18 cells.** The hydrodynamic diameter of U87- (A-i) and LN18- (B-i) derived CmEVs. Flow cytometric analysis of the expression of tetraspanins (CD9, CD63, CD81) on the membrane of CmEVs derived from U87 (A-ii) and LN18 (B-ii) cells. Comparison of typical CmEV (TSG101, CD9) and endoplasmic reticulum (ER) markers (GRP-94) detected by immunoblotting in whole cell lysates (WC) and lysates of CmEVs derived from U87 (A-iii) and LN18 (B-iii) cells. Percentage of PKH-positive CmEVs in U87 (A-iv) and LN18 (B-iv) cells. Confocal images representing the co-localization of PKH-labelled CmEVs (green) in LDs and different organelles (lysosomes, endoplasmic reticulum, mitochondria) in U87 (small LDs, C) and LN18 (large LDs, D) cells. LDs and organelles are depicted in red, and nuclei are shown in blue (DAPI).

Free FF primarily enters GBM cells by diffusion through lipid membranes. In LN18 cells, FF is rapidly trapped in large LDs after internalization, whereas the uptake of FF into small LDs in U87 cells is much less pronounced. To avoid the uptake of free FF into large LDs, we introduced lipid-based nanocarriers as vehicles for delivering FF into LN18 cells. Uptake of lipid-based nanocarriers into GBM cells is predominantly based on endocytosis and, therefore, differs from the uptake of small hydrophobic molecules such as free FF. Compared to synthetic nanocarriers, cancer membrane-derived extracellular vesicles (CmEVs) exhibit several advantages, including unique targeting features stemming from their homologous binding properties. CmEVs are derived from autologous cancer cell membranes that can be formed by their self-assembly capacity and carry receptors that facilitate homologous signaling and targeted accumulation in the GBM TME [28,30,31,34]. The extracted cell membranes of U87 and LN18 cells were used to form vesicles with an average hydrodynamic diameter of ∼200 nm through an extrusion-/plunger technique (Avani lipid extruder with 200 nm filter) (Fig. 4A-i and B-i). Phenotypic characterization of the derived CmEVs by flow cytometry revealed the expression of the tetraspanins CD9, CD63, and CD81 in both, LN18- and U87-derived CmEVs (Fig. 4A-ii and B-ii). We validated the expression of CD9 and TSG101, which are typical EV markers, using immunoblotting and compared the results to those of whole cell lysates (Fig. 4A-iii and B-iii). The purity of the U87- and LN18-derived CmEVs was demonstrated by the absence of the glucose-regulated protein 94 (GRP-94), which is constitutively expressed only in the ER (Fig. 4A-iii and B-iii).

To study the intracellular trafficking of U87- and LN18-derived CmEVs after internalization in U87 and LN18 cells, freshly produced CmEVs were stained with the green fluorescent lipophilic membrane dye PKH67 (PKH), and the staining intensity was validated by flow cytometric analysis (Fig. 4A-iv and B-iv). We visualized intracellular trafficking into different cellular compartments after 24 h at 37 °C by confocal microscopy in U87 and LN18 cells after co-staining with their respective PKH-labelled CmEVs and fluorescence-labelled markers for lysosomes, mitochondria, LDs and ER. A quantification of the colocalization of markers specific for different subcellular organelles and FF-loaded CmEVs revealed that most FF-loaded CmEVs accumulated in the lysosomal compartment of LN18 cells (Supplementary Fig. S6). In contrast to free FF, no obvious accumulation of LN18-derived FF-loaded CmEVs was detected in large LDs, ER and mitochondria of LN18 cells (Supplementary Fig. S6). In U87 cells the FF-loaded CmEVs appeared to be equally distributed in lysosomes and mitochondria (Supplementary Fig. S6).

**Fig. 5:**
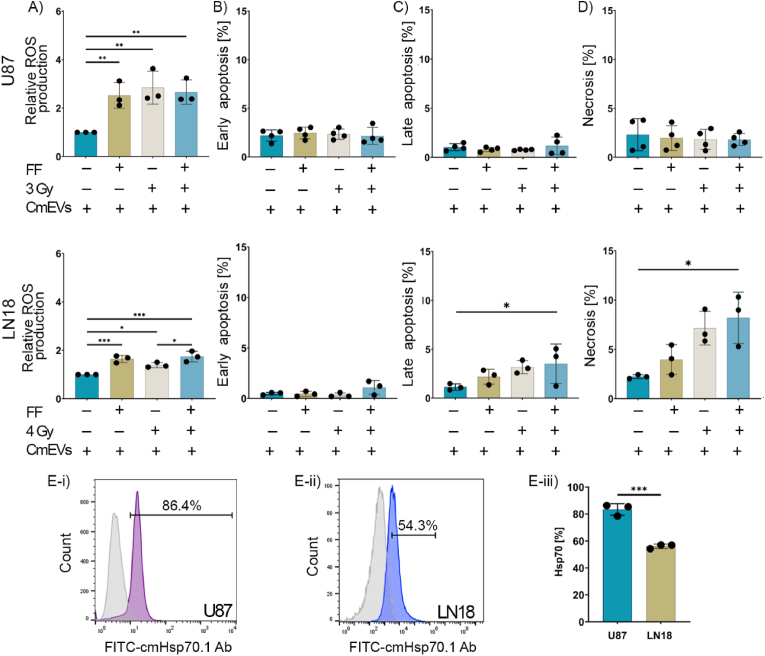
**Effect of fenofibrate (FF)-loaded and empty cancer membrane-derived extracellular vesicles (CmEVs) on the radiosensitivity of U87 and LN18 cells**. A) Relative reactive oxygen species (ROS) production in U87 and LN18 cells after treatment. B-D) Percentage of early apoptosis (B), late apoptosis (C), and necrosis (D) in U87 (top) and LN18 (bottom) cells after treatment with FF-loaded and empty CmEVs and irradiation (RTx). E-i/ii) Histograms show the expression of membrane Hsp70 on U87 and LN18 cells. Colored histograms represent cmHsp70.1-FITC positively stained cells, grey histograms show the staining pattern of an isotype-matched control antibody. E-iii) Mean percentage of membrane Hsp70 positivity in U87 and LN18 cells. Data represent mean values ± SD of three independent experiments. P values were calculated using unpaired *t*-test to compare two groups. ∗p < 0.05, ∗∗∗p = 0.0002 (E-iii), two-way analysis of variance (ANOVA) with Tukey's correction was used to compare more than two groups. ∗p = 0.0332, ∗∗p = 0.0021, ∗∗∗p = 0.0002.

Since large LDs in LN18 cells act as a sink for the free drug FF, which results in radioprotection, we explored the radiosensitizing potential of FF loaded in biomimetic CmEVs with different uptake and trafficking properties. We ensured that the functionality of FF-loaded CmEVs was equivalent to free FF; empty CmEVs were used as controls (data not shown). We then administered both FF-loaded CmEVs and empty CmEVs (control) to U87 and LN18 cells and exposed cells to the same single irradiation dose (3 Gy, 4 Gy, respectively) as used for the free drug. ROS levels were found to be significantly elevated in all treatment groups that received FF-loaded CmEVs, irrespective of the exposure to radiation when compared to control (Fig. 5A). Despite a significant increase in ROS levels in U87 cells after treatment with FF-loaded CmEVs, cell death (early apoptosis, late apoptosis, necrosis) was not significantly increased (Fig. 5B-D). In contrast, an increase in late apoptosis (∼5 %) and necrosis (∼10 %) was observed in LN18 cells after treatment with FF-loaded CmEVs and RTx (combi-treatment) (Fig. 5C and D). Since CmEVs predominantly accumulated in the lysosomal compartment of LN18 cells after uptake (Fig. 4C and D, Supplementary Fig. S6), we speculate that CmEVs might affect lysosomal stability and integrity.

Due to the high affinity of the major stress-inducible heat shock protein 70 (Hsp70) to tumor-specific glycosphingolipids, Hsp70 is not only expressed on the plasma membrane but also on lysosomal membranes of tumor cells, where it stabilizes membrane integrity [46]. Since the expression of Hsp70 on the plasma membrane reflects the expression of Hsp70 on lysosomal membranes, the expression of Hsp70 on the cell surface of viable U87 and LN18 cells was determined by flow cytometry. The data show that the Hsp70 membrane expression on U87 cells is significantly higher (86.4 %) than on LN18 cells (54.3 %) (Fig. 5E-i/ii/iii). Therefore, U87 cells might be more resistant to necrotic cell death than LN18 cells because of a higher stability of their lysosomal membranes after uptake of FF-loaded CmEVs. In summary, treatment with FF-loaded CmEVs as a functional nanocarrier might improve radiosensitivity in tumor cells with large LDs and a low membrane Hsp70 expression.

## 3 Discussion

One of the hallmarks of cancer is abnormal lipid metabolism [47] which induces the formation of lipid droplets (LDs) that promote tumor survival and radiation resistance. In this study, we wanted to overcome the radiation resistance of GBM cell lines U87 and LN18 using the clinically applied lipid-lowering drug FF in different formulations and to investigate its mechanism of action. Although it is already known that FF negatively affects cell viability in different tumor cell types [[48], [49], [50]], its radiosensitizing capacity has not yet been studied in GBM. In line with previous findings, concentrations of FF above 50 μM and 100 μM significantly impaired the viability of U87 and LN18 tumor cells, respectively (Supplementary Fig. S1) [50]. Interestingly, a combined treatment consisting of a sub-lethal concentration of FF and irradiation radiosensitizes U87 cells, but radioprotects LN18 cells (Fig. 1A). In human ECA-109 and TE-13 esophageal carcinoma cells, a radiosensitizing effect of FF has previously been linked to a G_2_/M cell cycle arrest and reduced VEGF expression [51], whereas radioprotection of normal skin fibroblasts (HaCat) by FF was associated with an upregulation of fatty acid binding protein 4 (FABP4), facilitating the FA transport from the surrounding environment [52]. In our GBM cell lines, the cell cycle appeared not to be affected by a treatment with FF at different concentrations (data not shown).

Ionizing radiation induces DNA damage [6], resulting in DNA double-strand breaks that can be measured by the formation of γH2AX-DNA complexes [53,54]. Accordingly, a further increase in the γH2AX-DNA complex formation 30 min after FF pre-treatment and irradiation (3 Gy) was only observed in U87 cells, whereas in LN18 cells, γH2AX-DNA complex formation decreased slightly after a combined treatment regimen (FF and 4 Gy) (Fig. 1B). More than 70 % of the RTx-induced DNA double-strand breaks that cause apoptosis are indirectly caused by ROS, predominantly originating from mitochondria [6,55]. Irradiation of U87 and LN18 cells with 3 Gy and 4 Gy, respectively, increased ROS production in both cell lines. However, we only observed a further increase in ROS levels after pre-treatment with FF (Fig. 1C) concomitant with a decrease in MMP (Fig. 1D) and a significant increase in early apoptotic cells (Fig. 1E) in U87 cells but not in LN18 cells. This finding is supported by earlier studies showing that elevated ROS levels can damage mitochondria, impair their oxidative capacity, and ultimately trigger irreversible apoptotic cell death [56], whereas lower ROS levels are associated with improved cell survival [57]. In addition to DCFDA (2′,7′-Dichlorofluorescin diacetate) which detects hydrogen peroxide, hydroxyl radicals, carbonate radicals and nitrogen dioxide, Dihydroethidium (DHE) which detects cytosolic superoxide, hydroxyl radicals and peroxynitrite was used to detect different ROS species [36]. Both tests showed the same trend in U87 and LN18 cells following treatment with FF, RTx and a combination of both treatments (Supplementary Fig. S2).

We then studied the lipid metabolism in U87 and LN18 cells in more detail, to investigate the mechanisms responsible for the contradictory radiosensitizing/radioprotective effects of FF in these cell lines. Cancer cells typically accumulate LDs that contribute to cell death resistance [47] by storing excessive FA to protect them from lipotoxicity and redox imbalance especially in a hypoxic environment [19,21,22,58]. Lipids are produced either by *de novo* lipid synthesis mediated by FASN, a key enzyme in the initial stages, or by exogenous lipid uptake via CD36, a scavenger receptor expressed on the cell surface of different cell types (Fig. 2A). The cell surface expression of CD36, which enhances the uptake of lipids from the TME and the oxidation of FA to TAG [59], was significantly higher in LN18 cells than in U87 cells (Fig. 2B). Under hypoxic, but not normoxic conditions, other FA transporters, such as fatty acid binding proteins FABP3 and FABP7 are important for the growth of GBM [60]. The transfection of FABP7 promotes migration and infiltration of U87 cells [61]. Since under normoxic conditions FABP3 and FABP7 in untreated and FF/RTx treated U87 and LN18 cells remained under the detection level (data not shown), we speculate that these FABPs might not play an essential role in the radiosensitizing/radioprotective effects of FF on small and large LDs in these cell lines. However, the expression of the fatty acid transporter CD36 is significantly higher in LN18 cells compared to U87 cells. Therefore, we postulate that CD36 is responsible for the accumulation of large LDs in LN18 cells which mediate radioprotection in these cells upon treatment with the free drug FF.

The enzyme DGAT1 promotes the production of small LDs to avoid lipotoxicity [56]. U87 cells exhibited higher basal DGAT1 levels than LN18 cells (Fig. 2D-ii) which is associated with the larger number of small LDs in U87 cells (Fig. 2C) and irradiation results in a further increase in the numbers of small LDs in U87 cells (Fig. 2E and F). However, a treatment with FF alone or in combination with irradiation significantly reduced the expression of DGAT1 in U87 cells (Fig. 2G) and decreased the number of irradiation-induced elevated numbers of small LDs (Fig. 2E and F). Previous studies have shown that targeting DGAT1 in GBM or prostate cancer can impair FA metabolism and thus induce mitochondrial damage, excessive ROS production, and ultimately apoptosis [56] through a blockade of the autophagy flux [62]. We demonstrate that the clinically applied lipid-lowering drug FF can induce lipotoxicity in U87 cells by inhibiting the production of small LDs via DGAT1 and by increasing the oxidative stress. Nevertheless, the treatment did not affect the FA transporter CD36 and other FA-binding proteins such as FABP3 and FABP7 remained under the detection level in both cell lines, irrespective of the treatment (data not shown).

In contrast to U87 cells, a combined treatment with FF and irradiation significantly increased the number of large LDs in LN18 cells (Fig. 2E and F). This increase in large LDs was accompanied by a significant increase in the DGAT1 expression (Fig. 2G) and was associated with an FF-dependent upregulation of GPAT4, an ER-residing enzyme that promotes the formation of large LDs (Fig. 2H). We assume that the accumulation of large LDs in LN18 cells after FF pre-treatment might be linked to radiation resistance in LN18 cells. This finding is supported by studies showing that radiation therapy promotes recurrence in GBM because of an accumulation of FA in large LDs, thereby preventing ER stress and reducing apoptosis [23]. We assume that the highly lipophilic environment enabled by the presence of large LDs promoted by GPAT4 in LN18 cells allows the absorption of the hydrophobic drug FF and thereby inhibits its radiosensitizing capacity. In line with these findings we could demonstrate that the presence of large LD induced by OA in U87 cells (Fig. 3A) mediates radioprotective effects in U87 cells upon FF treatment similarly to LN18 cells, although U87 cells containing small LDs can be radiosensitized by FF (Fig. 3B-F). This indicates that the presence of large LDs in an autologous cell system is mediating radioresistance in GBM. A small limitation of the study was that we could not induce an overexpression of GPAT4 in U87 cells to show a direct link between GPAT4, large LDs and radioresistance. The importance of the lipid metabolism in drug resistance is further supported by the finding that a pharmacological inhibition of LD formation increases the effectiveness of curcumin, a drug with lipophilic properties that enhances the killing of U251 N GBM cells via inhibition of cPLAα [45].

To avoid the radioprotective effect of free FF, we encapsulated FF (at equivalent amounts as used in free FF experiments) in LN18 and U87 cell membrane-derived extracellular lipid nanoparticles (CmEVs), which were used as a vehicle for the drug. In contrast to hydrophobic free FF, which is internalized by diffusion, CmEVs are endocytosed by tumor cells, triggering different intracellular trafficking and accumulation processes. In LN18 cells, endocytosed FF-loaded biomimetic CmEVs accumulated predominantly in the lysosomes but not in large LDs (Fig. 4C and D, Supplementary Fig. S6). This enabled FF to stimulate lysosomal proteases, which orchestrate lysosomal membrane permeabilization [63] and tumor cell necrosis (Fig. 5D). Comparing the radiosensitizing effects of free FF and encapsulated FF revealed that U87 cells are predominantly killed by apoptotic cell death caused by free FF via oxidative stress induced by MMP impairment, whereas LN18 cells are killed by necrosis caused by the encapsulated drug via lysosomal membrane disintegration (Fig.1 C-E) (Fig. 5A-D).

It is important to note that the stability of plasma and lysosomal membranes depends on the amount of Hsp70 in the membrane. Hsp70, which has a high affinity to tumor-specific glycosphingolipids, is known to act as a stabilizer for the lysosomal and plasma membranes of tumor cells [46]. The expression of plasma membrane-bound Hsp70, which reflects the expression of Hsp70 on lysosomal membranes, was significantly higher in U87 cells than in LN18 cells. Thus, we can assume that the lysosomal membranes of U87 cells are more resistant to damage than those of LN18 cells. This finding might explain the observed differences in cell death between the cell lines after treatment with FF-loaded CmEVs (Fig. 5C and D). LN18 cells were more sensitive to lysosomal damage after uptake of FF-loaded CmEVs and underwent necrotic cell death upon irradiation. In contrast, U87 cells containing small LDs underwent apoptotic cell death after combined treatment with free FF and radiation.

**Fig. 6:**
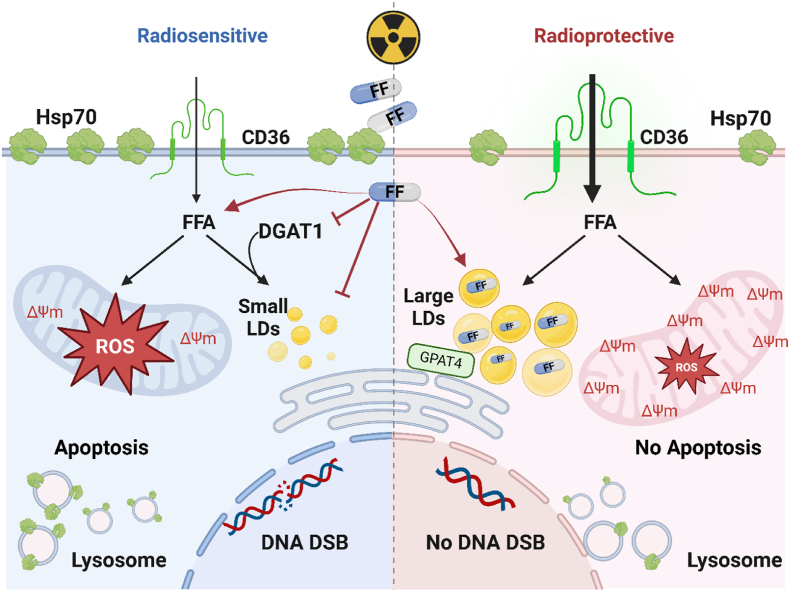
**Proposed mechanisms for the radiosensitizing and radioprotective effects of fenofibrate (FF) observed after combined treatment (FF and radiation) in U87 and LN18 cell lines.** Free FF induces radiosensitivity in glioblastoma cells with small lipid droplets (LDs) primarily through increasing DNA double-strand breaks (DSB). FF inhibits DGAT1 expression, leading to a reduction in LDs coupled with an increase in the accumulation of reactive oxygen species (ROS) and disturbance of mitochondria homeostasis, resulting in a reduction of mitochondrial membrane potential (shown by Δψm) and apoptosis. Large LDs produced by GPAT4 can sequester free FF, reducing its availability and effectiveness and enhancing the radioprotective effect. FFA – free fatty acids.

**Fig. 7:**
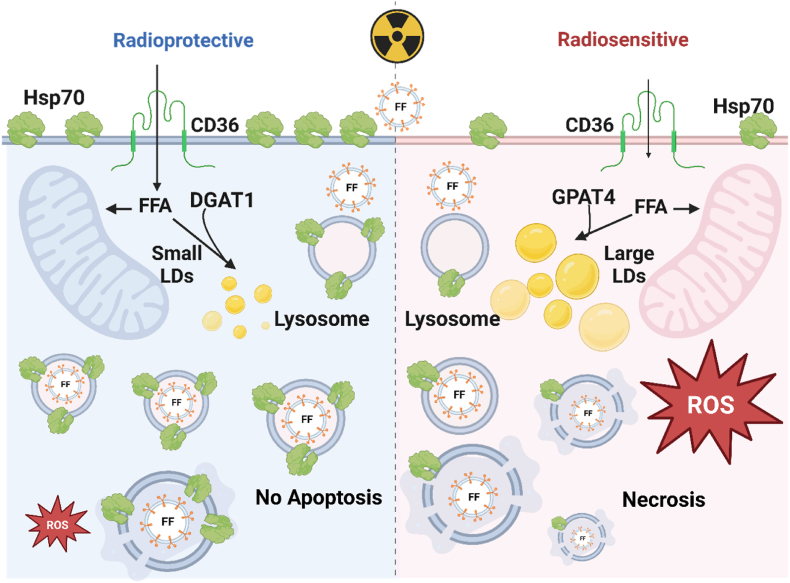
**Proposed mechanisms for the radiosensitizing and radioprotective effects of fenofibrate (FF)-loaded cancer membrane-derived extracellular vesicles (CmEVs) in U87 and LN18 cell lines.** In glioblastoma (GBM) cells expressing high levels of membrane Hsp70, treatment with FF-loaded-CmEVs elicits a radioprotective effect. Conversely, in GBM cells with large LDs, FF-loaded-CmEVs enhance radiosensitivity by increasing reactive oxygen species (ROS) levels and inducing necrosis via decreased lysosomal stability. The sensitization effect depends on lysosomal stability; FF-loaded-CmEVs induce lysosomal stress, causing lysosome swelling and permeabilizing the lysosomal membrane. FFA – free fatty acids.

In summary: i) the lipid metabolism of GBM cells is involved in the formation of small and large LDs (Fig. 6); ii) small and large LDs determine whether FF as a free drug or FF encapsulated in CmEVs can radiosensitize GBM cells (Fig. 7); iii) the amount of Hsp70 which is responsible for the membrane integrity in plasma and lysosomal membranes determines the sensitivity of GBM cells to necrotic cell death after irradiation. Taken together, our results provide a promising avenue to improve the efficacy of radiotherapy in GBM by FF as a free drug or in liposomes, potentially allowing for lower radiation dosages without compromising treatment outcomes, and thereby improving the quality of life for patients.

## 4 Conclusions

In this study, we showcase the potential of FF as a radiosensitizing drug to improve radiotherapy outcomes in GBM patients and reduce the radiation dose required using two GBM cell lines. We observed that FF can exhibit radiosensitizing or radioprotective effects associated with small and large LDs, respectively (Fig. 6). The development of these LDs depends on differences in lipid metabolism in the two GBM cell lines. Significantly, this finding emphasizes the importance of investigating differences in the lipid metabolism of tumor cells to ensure whether a treatment would be beneficial or harmful for a patient. We also demonstrate that changing the formulation of a drug to alter its uptake mechanism and intracellular trafficking can affect its efficacy as a radiosensitizer (Fig. 7). Thus, a better understanding of a tumor's lipid metabolic state can enable the development of novel strategies to overcome treatment resistance, moving towards personalized medicine and improved patient outcomes.

## 5 Materials and methods

**Cell culture.** Human GBM cell lines U87 and LN18 were maintained in high glucose Dulbecco's Eagles's Minimum Essential Medium (DMEM) (Sigma-Aldrich) containing 10 % v/v heat-inactivated fetal bovine serum (FBS) (Sigma-Aldrich) and 1 % v/v antibiotics (10,000 IU/mL penicillin and 10 mg/mL streptomycin, Sigma-Aldrich). All cells were maintained at 37 °C and 5 % v/v CO_2_ atmosphere and tested regularly for mycoplasma contamination.

**Reagents and treatment.** FF stock was prepared by dissolving powdered FF in dimethyl sulfoxide (DMSO) (Sigma-Aldrich) at a concentration of 100 mM. The resulting solution was stored at −20 °C for future use. This solution was diluted into designated concentrations in the same culture medium as used in experiments, ensuring a constant DMSO concentration of 0.1 % v/v in each group. Cells were incubated with a sublethal concentration of FF or EVs for 24 h, followed by exposing the cells to irradiation with a single dose of 0 Gy (sham), 3 Gy or 4 Gy x-rays using CIX2 RS225 (XStrahl) using 0.5 mm cobalt filter at a dose rate of 1 Gy/45 s and 40 cm distance from the source (15 mA, 195 kV). The experiments were conducted 24 h post-irradiation (48 h after the initial FF or cmEV treatment).

**Viability assay.** Cells were seeded in a 96-well plate with a density of 10000 cells/well and treated with increasing concentrations of FF (0, 12.5, 25, 50, 100 μM). After 24 h, the medium was replaced with CellTiter-Glo® diluted 1:1 with fresh medium, after which cells were incubated on a shaker for 2 min to induce cell lysis. Next, the plate was placed at 37 °C for 10 min in the dark before the luminescent signal was recorded by the plate reader. The luminescent signal value of all conditions was normalized to the control (0 μM) after background subtraction.

**Colony formation assay.** Cell colony formation ability was assessed using a plate-colony formation assay. For U87 cells, a total of 300, 400, and 500 cells were seeded in 6-well plates with 2 mL of complete DMEM medium. Each well plate was treated with 0, 25, and 50 μM FF for 24 h (2 wells per FF dose). The 6-well plates were then subjected to radiation doses of 0 Gy (300 cells), 2 Gy (400 cells), and 4 Gy (500 cells). For LN18 cells, a total of 100, 250, and 500 cells were seeded in 12-well plates with 1 mL of complete DMEM medium, followed by FF treatment (0, 25, and 50 μM; 4 wells each) for 24 h, then subjected to 0, 2, and 4 Gy, respectively. Cells were cultured for 10 days, after which colonies were fixed with cold pure methanol for 5 min, stained with 0.1 % w/v crystal violet solution for 3 min, and air-dried. The colonies (at least 50 cells) were counted using an automatic colony counter Bioreader® 3000 (Bio-Sys GmbH). Survival curves were fitted to the linear quadratic model using GraphPad Prism 9.

**DCFDA cellular ROS assay.** A Cellular ROS detection Assay Kit (Cat: ab113851, Abcam) was employed to detect ROS species following the manufacturer's instructions. Following the treatment regimen, 0.2x10^6^ cells were stained with a 2′,7′-Dichlorofluorescin diacetate (DCFDA) probe (20 μM) for 30 min at 37 °C. Fluorescence intensity was then measured at 495/529 nm using a FACSCalibur™ flow cytometer (BD Biosciences). The ROS levels of cells were analyzed using FlowJo™ data analysis software (v10.10).

**DHE (Dihydroethidium) Assay Kit.** ROS were determined in U87 and LN18 cells using the DHE ROS kit (Cat: ab236206, Abcam) following the manufacturer's instructions. Briefly, treated and untreated cells were washed once with cell base buffer and then stained with DHE (1:1000) for 1 h at 37 °C. After another washing step with cell base buffer cells were analyzed on a FACSCalibur™ flow cytometer using the PE channel. The ROS levels were analyzed using FlowJo™ data analysis software (v10.10). All data were normalized to the control group.

**Western blot analysis.** Cell pellets were lysed in radioimmunoprecipitation assay (RIPA) buffer mixed with protease cocktail tablets (cOmplete tablets, Roche Diagnostic GmbH) and phosphatase inhibitors (PhosphoSTOP, Roche Diagnostic GmbH). EVs were homogenized with reducing reagents only. A Pierce™ BCA Protein Assay Kit (Thermo Fisher Scientific) was used to determine the protein content. The proteins (20 μg) were separated on an SDS-PAGE (8–15 % w/v) and transferred onto activated polyvinylidene fluoride membranes (PVDF). Membranes were blocked using a 1x Roti block buffer for 1 h at room temperature (RT). Primary antibodies were diluted in the same buffer and incubated at 4 °C overnight (Table S1). Membranes were stained with the appropriate secondary antibody (Table S1) and 1x Roti block buffer for 60 min at RT. A Pierce™ ECL Western Kit (Thermo Fisher Scientific) was used to visualize the protein bands. Pictures were acquired using a ChemiDoc™ Touch Imaging System (Bio-Rad). The protein expression ratio for each sample was quantified using ImageJ Software.

**Flow cytometry.** Cells (0.25x10^6^) were trypsinized, washed twice with ice-cold flow cytometry buffer (phosphate-buffered saline (PBS; Life Technologies)), containing 10 % v/v FBS (Sigma-Aldrich) and incubated with FITC-conjugated CD36 monoclonal antibody (mAb, AC106, 2 μg/mL, Miltenyi Biotec), and membrane Hsp70 mAb (cmHsp70.1, 20 μg/mL, multimmune GmbH) for 30 min in the dark on ice. For intracellular staining, cells were trypsinized and fixed using a fixation buffer for 60 min at RT, followed by permeabilizing the cells in 1:10 permeabilized buffer containing the GPAT4 (Cell Signaling Technology) antibody in a dilution of 1:2000 for 60 min at RT. Cells were then washed with flow cytometry buffer and centrifuged for 5 min at 1200 × *g*. Cells stained with FITC secondary antibody (Alexa Fluor® 488 AffiniPure™, Jackson ImmunoResearch) were diluted in PBS (1:10000) for 60 min at RT. After a further wash step, the expression of each membrane receptor or intracellular protein was determined using a FACSCalibur™ flow cytometer (BD Biosciences) and the data analyzed using FlowJo™ data analysis software (v10.10). For membrane receptor detection, propidium iodide (PI, 1 μg/mL, Sigma-Aldrich) was added immediately before flow cytometric analysis to exclude non-viable cells from the analysis. Only viable (PI-negative) cells with intact cell membranes were gated on and analyzed. IgG1 isotype-matched FITC-labelled immunoglobulin (mouse IgG1-FITC, 345815, BD Biosciences) was used to assess nonspecific binding. The positivity was determined by subtracting the percentage of cells stained with the isotype-matched control antibody from the percentage of cells positively stained with the monoclonal antibody.

**Detection of apoptosis. Annexin V-FITC apoptosis staining/detection.** Apoptotic cells were detected by an Annexin V-FITC apoptosis staining kit (ab14085, Abcam). As instructed by the manufacturer, 2.5x10^5^ cells were resuspended in 250 μL of Binding Buffer II/1X Binding Buffer, 2.5 μL Annexin V-FITC, and 2.5 μL of propidium iodide (PI). Then, the treated cells were incubated for 5 min in the dark at RT before being analyzed using a FACSCalibur™ flow cytometer (BD Biosciences) and FlowJo™ data analysis software (v10.10). Cells positively stained for Annexin V-FITC are considered as early apoptotic, cells positively stained for Annexin V-FITC and PI are considered as late apoptotic. Cells positively stained for PI are considered as necrotic.

**Detection of mitochondrial membrane potential (MMP).** MMP was determined using the TMRE-Mitochondrial Membrane Potential Assay Kit (ab113852, Abcam). For this, cells were seeded in a T25 flask at a density of 0.3x10^6^ and treated as described above. The treated cells were rinsed with washing solution (PBS and BSA 0.2 % w/v) once and then incubated for 30 min with tetramethylrhodamine ethyl ester (TMRE, 200 nM) which was diluted in the washing solution at 37 °C and 5 % v/v CO_2_. The treated cells were washed twice with the washing solution, trypsinized, and resuspended in flow cytometry buffer. The fluorescent intensity was detected at Ex/Em 549/575 nm using a FACSCalibur™ flow cytometer (BD Biosciences) and the data analyzed using FlowJo™ data analysis software (v10.10).

**Lipid droplet staining.** Cells grown on a glass coverslip were treated with FF and radiation using the same protocol as previously described. Following treatment, the cells were fixed with 3.6 % w/v paraformaldehyde for 30 min at RT. Subsequently, LDs were stained with 20 μM HCS LipidTOX™ Deep Red Neutral Lipid Stain (Thermo Fisher Scientific) for 30 min at RT in the dark. After staining, the cells were rinsed 3 times with PBS, and the nuclei were stained with Hoechst33342 (1:1000) for 15 min at RT. To stain GPAT4 on the LD surface, cells were incubated with the primary antibody against GPAT4 for 60 min at RT, followed by incubation with the appropriate secondary antibody. The cells were washed with PBS, mounted on a slide, and visualized using confocal microscopy. Images were analyzed with ImageJ software.

**Formation of FF-loaded CmEVs.** The CmEVs were prepared using an established protocol [32,64,65], taking advantage of the physical distribution of the cell membrane. Briefly, the trypsinized LN18 and U87 cancer cells were washed by PBS, resuspended in a hypotonic lysing buffer, which included cOmplete protease inhibitor (1X), and disrupted using a Dounce homogenizer or ultrasonicator. The solution was centrifuged at 500 × *g* for 5 min and 12000 × *g* for 15 min to remove the unruptured cells and intracellular compartments. The supernatant was collected and centrifuged at 100,000 × *g* for 120 min. The resulting pellets were collected, washed with PBS, and mixed with PBS into a solution, including cOmplete protease inhibitor without or with FF 25 μM. The solution was then sonicated for 5 min, extruded through 200 nm polycarbonate porous membranes using a mini extruder (Avanti Polar Lipids), and concentrated using an Amicon 10 kDa MWCO filter column (3000 × *g* for 20 min). The hydrodynamic size distribution of the formed CmEVs was measured using dynamic light scattering (DLS) on a Zetasizer instrument (Malvern, UK). The expression of tetraspanins (CD9, CD63, CD81) on the CmEVs was measured by flow cytometry using exosome capture beads according to the manufacturer's instructions. The presence of typical vesicular markers (e.g., CD9, TSG101) and the absence of ER-residing markers (e.g., GRP-94) were determined by Western blot analysis.

**FITC stained CmEVs.** To obtain FITC-labelled CmEVs (FITC-CmEVs without FF), the formed CmEVs were stained using a PKH67 green fluorescent cell linker kit (Sigma-Aldrich) according to the manufacturer's protocol. Briefly, the plate containing formed CmEVs was mixed with 1 mL of Diluent C and sonicated for 5 min. Next, PKH67 dye (5 μL) was added to the CmEVs/Diluent C mixture, mixed for 30 s by gentle pipetting, and incubated for 5 min at RT. The PKH67 dye in the mixture was quenched by adding 2 mL of 10 % w/v BSA in PBS, then slowly adding 1.5 mL of the 0.971 M sucrose solution to the bottom of the centrifuge tube, ensuring the exosome-dye solution remained on top. The mixture was then centrifuged at 100,000 × *g* for 120 min at 5 °C. Next, the stained pellet was resuspended in PBS by gentle pipetting and transferred to an Amicon 10 kDa MWCO filter column (3000×*g* for 20 min) to remove free dyes, after which they were analyzed by flow cytometry. The FITC-CmEVs were prepared and used fresh for all experiments.

**Intracellular trafficking of FITC-CmEVs.** U87 and LN18 cells were incubated with FITC-CmEVs for 24 h and then fixed with PFA (3.6 % w/v). Following this, organelles were stained according to the manufacturer's instructions and imaged with confocal microscopy imaging. Briefly, lysosomes were stained with LysoTracker™ Red DND-99 (1 μM, 30 min, Thermo Fisher Scientific), mitochondria were stained with MitoTracker™ Red (1 μM, 30 min, Thermo Fisher Scientific), and endoplasmic reticula were stained with ER-Tracker™ Red (1 μM, 30 min, Thermo Fisher Scientific). The colocalization of CmEVs in different subcellular organelles was performed by ImageJ using Person's coefficients.

**Statistical analysis.** All statistical analyses were performed using GraphPad Prism 9. Data are presented as mean ± standard deviation (SD) of at least three independent experiments. Differences between groups were assessed using a one-way analysis of variance (ANOVA) followed by Tukey's post-hoc test for multiple comparisons. For experiments involving more than two conditions, a two-way ANOVA was employed to evaluate the interaction between different factors, followed by Tukey's multiple comparisons test to identify significant differences between specific groups. Statistical significance was defined as a p-value of less than 0.05. In figures, significance levels were denoted as: ∗p < 0.0332, ∗∗p < 0.0021, ∗∗∗p < 0.0002 and ∗∗∗∗p < 0.0001.

## CRediT authorship contribution statement

**Bayan Alkotub:** Writing – original draft, Validation, Methodology, Investigation, Formal analysis, Conceptualization. **Lisa Bauer:** Methodology, Investigation. **Ali Bashiri Dezfouli:** Methodology. **Khouloud Hachani:** Methodology. **Vasilis Ntziachristos:** Supervision, Funding acquisition. **Gabriele Multhoff:** Writing – review & editing, Validation, Supervision, Funding acquisition, Conceptualization. **Morteza Hasanzadeh Kafshgari:** Supervision, Methodology, Investigation, Conceptualization.

## Declaration of competing interest

V.N. is a founder and equity owner of Maurus OY, sThesis GmbH, iThera Medical GmbH, Spear UG and I3 Inc. No conflicts of interest are declared by any of the other authors.

## Data Availability

Data will be made available on request.
