# Supplementary material for: Radiosensitizing capacity of fenofibrate in glioblastoma cells depends on lipid metabolism
Source: Redox Biol. 2024 Dec 2;79:103452. doi: 10.1016/j.redox.2024.103452 (PMC11697781; doi:10.1016/j.redox.2024.103452)
Supplement: Multimedia component 1 [file mmc1.docx]

**Radiosensitizing Capacity of Fenofibrate in Glioblastoma Cells Depends on Lipid Metabolism**


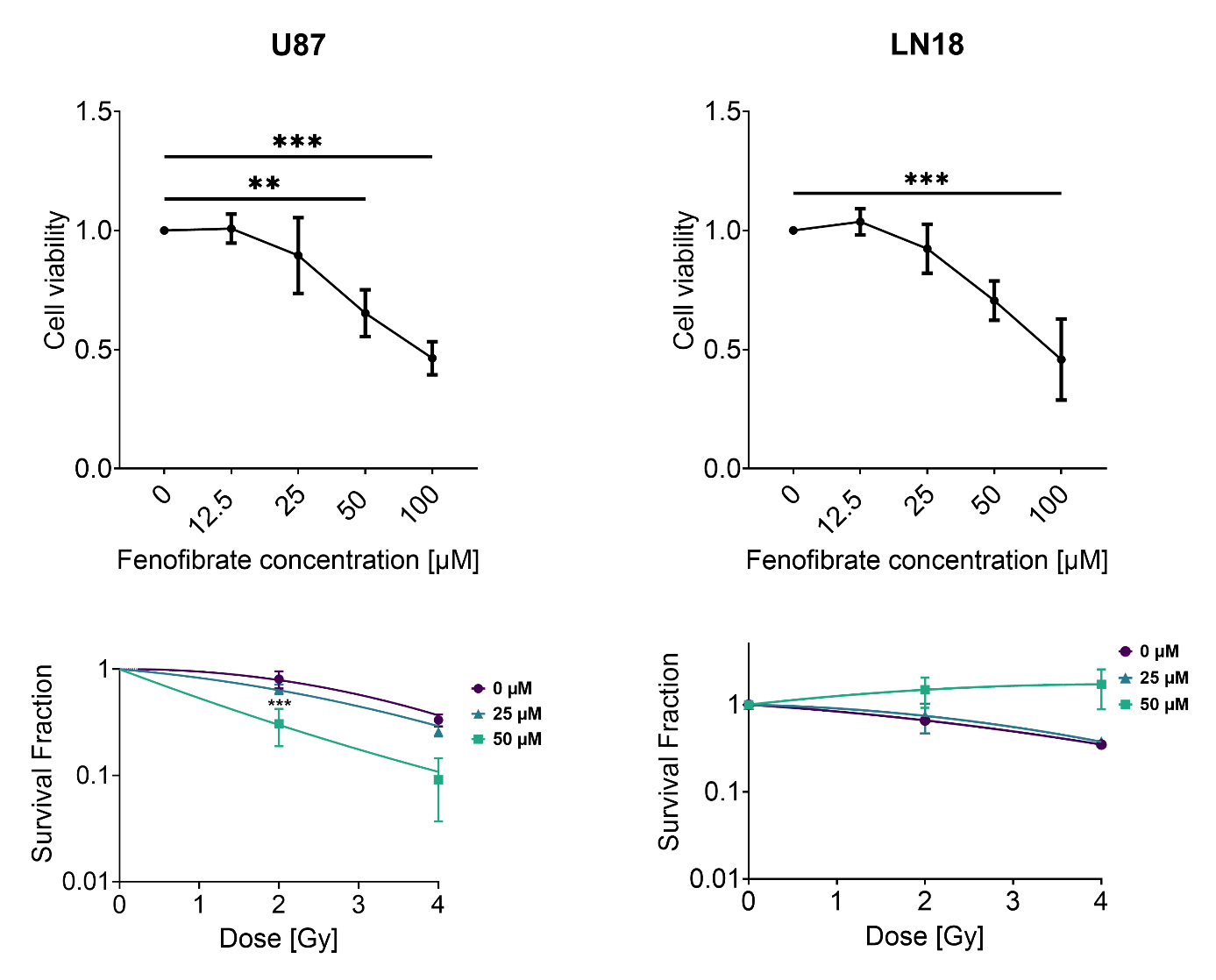


B

A

Figure S1. Effect of FF on cell viability of glioblastoma cells. (A) U87 and (B) LN18 cells were treated with increasing concentrations of FF (0, 12.5, 25, 50, 100 µM) for 24 h, and viability was measured using CellTiter-Glo assay. Cell viability was expressed as a luminescence signal relative to the luminescence signal of DMSO (control), which was considered as 1. Mean values and standard deviation (± SD) of 3 biological replicates (n=3); p values: *p ≤ 0.05, **p ≤ 0.005, ***p ≤ 0.0005 calculated by one-way ANOVA followed by Tukey's multiple comparison test.


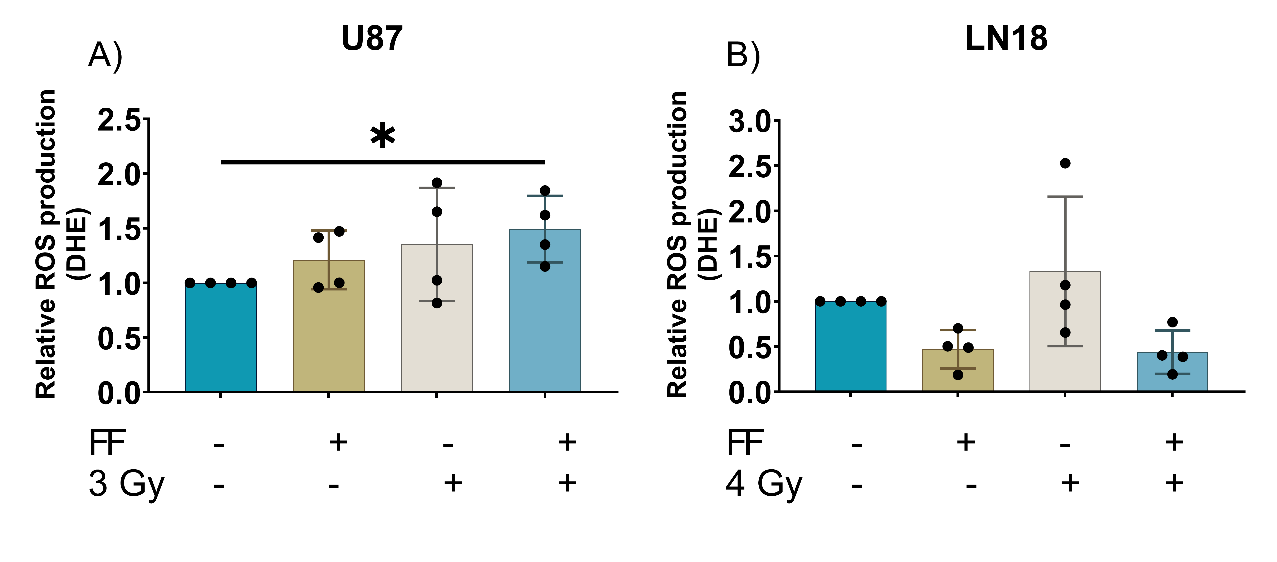


Figure S2. Effect of FF and RTx on ROS levels in U87 and LN18 cells using the DHE assay. A) Relative ROS production in A) U87 and B) LN18 cells 24 h after treatment with FF (25 µM), RTx (3 Gy for U87 cells, 4 Gy for LN18 cells), or a combined treatment. The ROS production was expressed as mean fluorescent intensity relative to control, which was considered as 1. Mean values and standard deviation (± SD) of 3 to 4 experiments (n=3-4); *p = 0.0332, calculated by two-way (ANOVA) with Tukey’s correction.


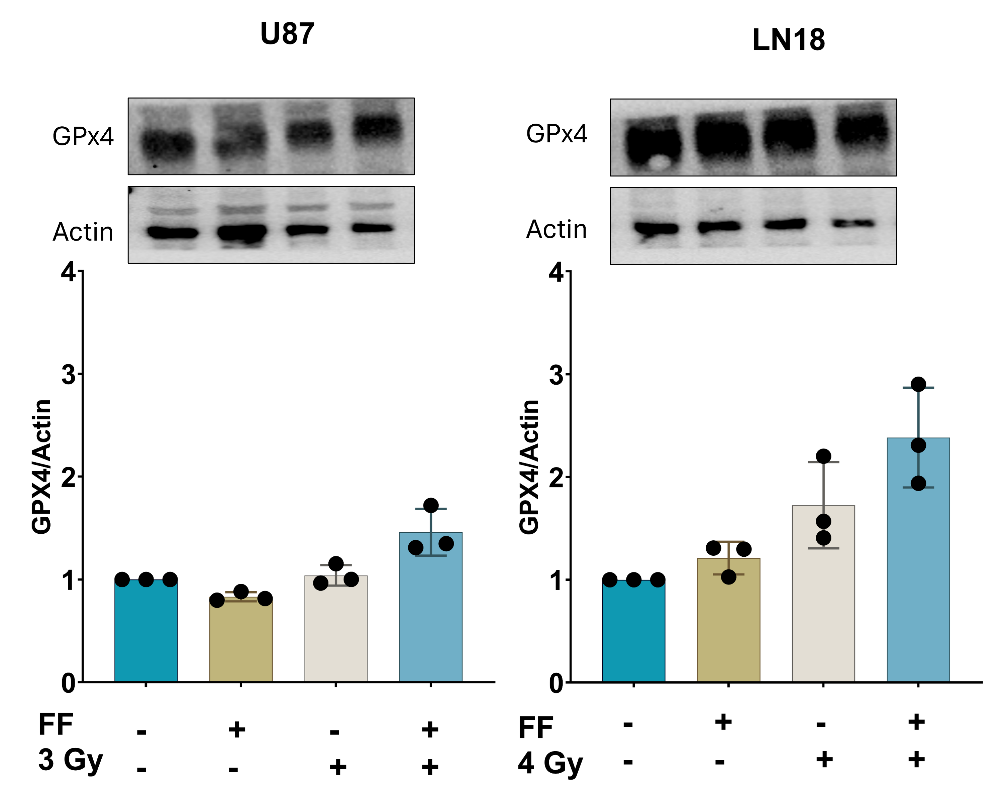


Figure S3. Effect of FF and RTx on the expression of the ferroptosis marker GPx4 in U87 (left panel) and LN18 (right panel). Cells were treated with FF (25 µM), irradiation (3 Gy for U87 cells, 4 Gy for LN18 cells) and a combined treatment. Actin was used as a loading control.
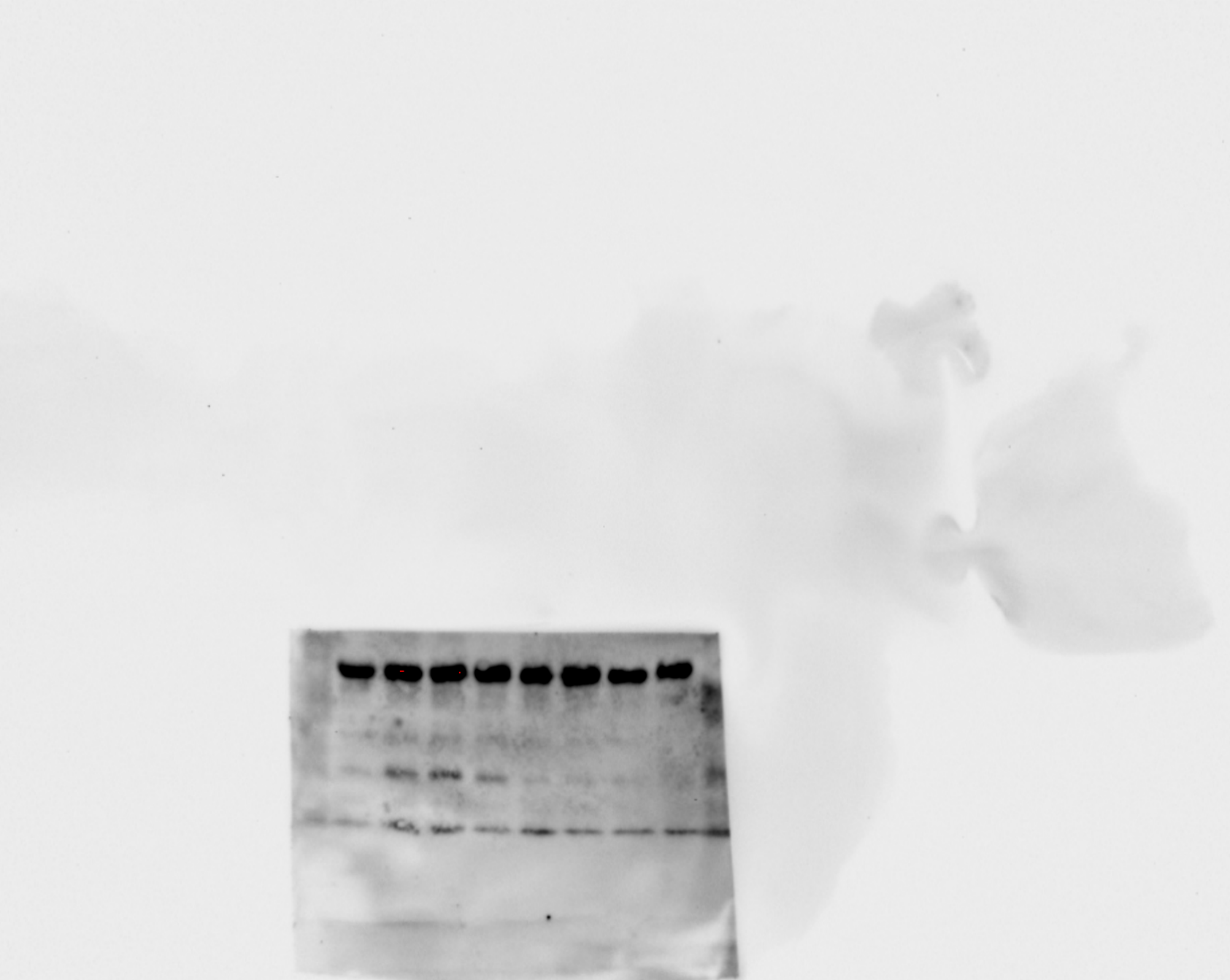

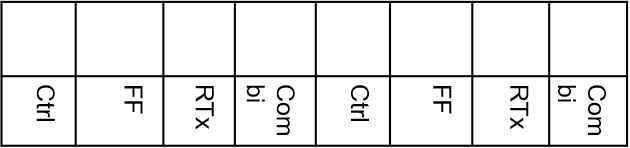

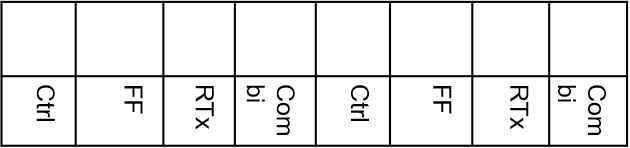

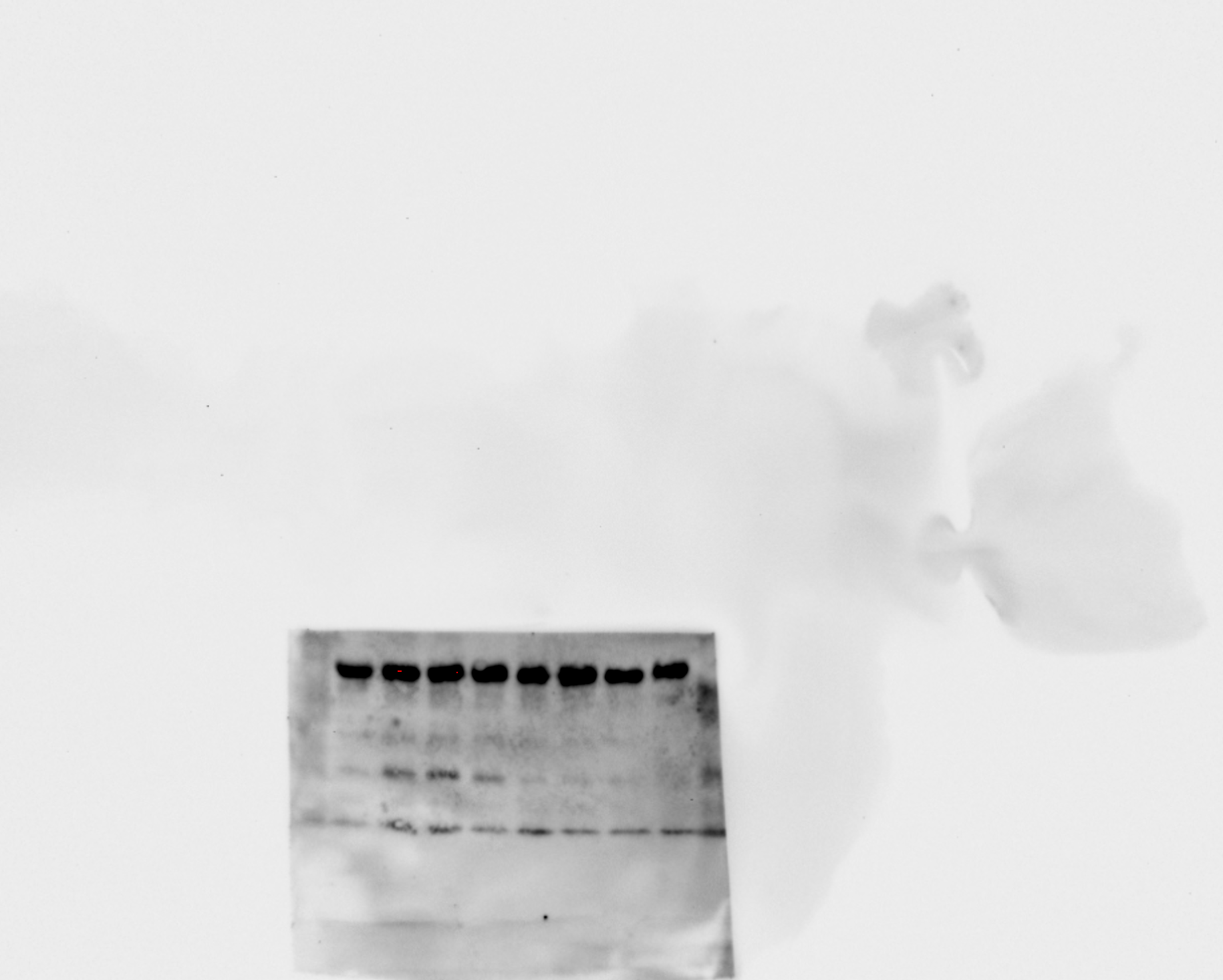


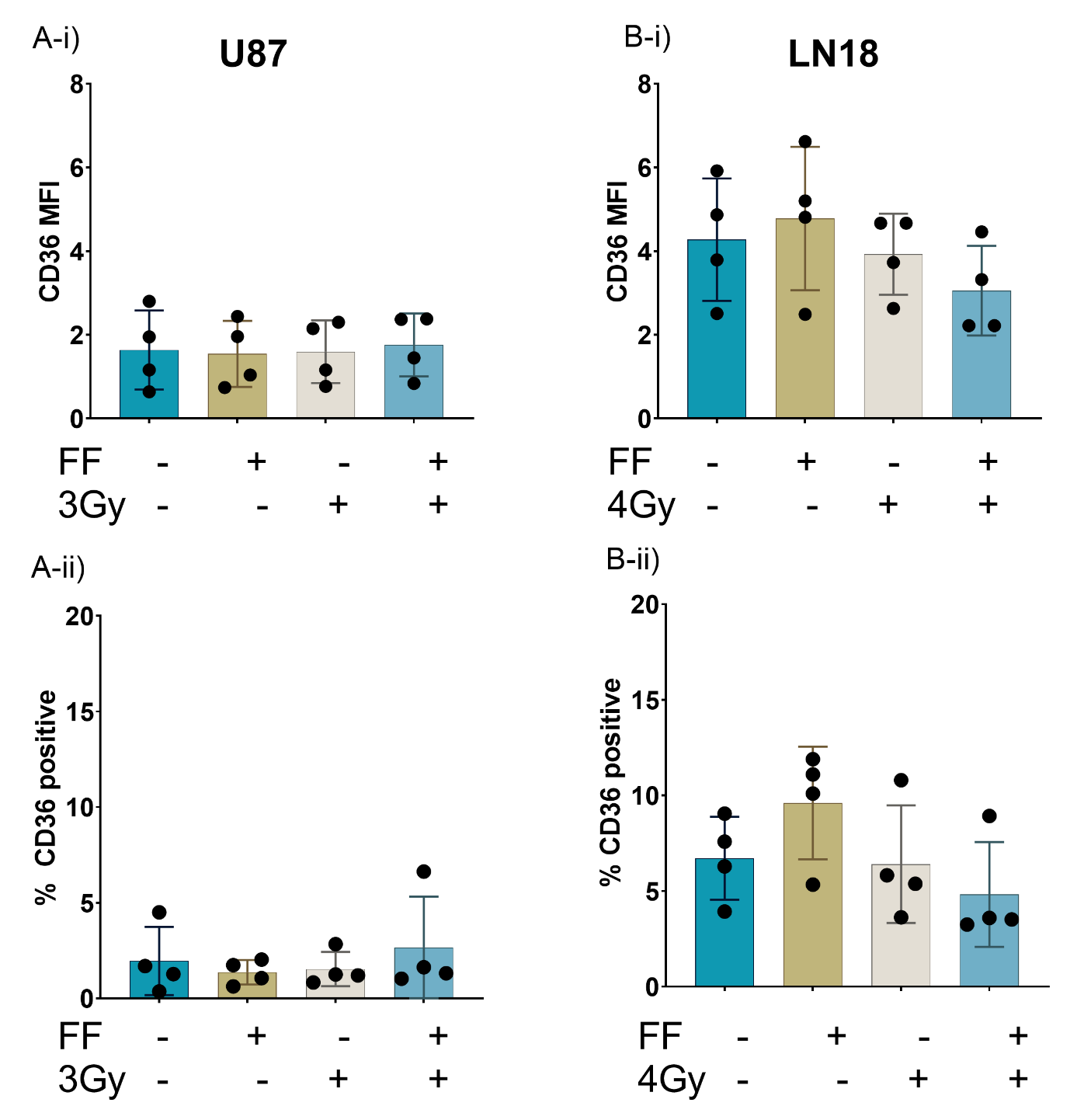


Figure S4. Effect of FF and RTx on the expression of the fatty acid transporter CD36 in U87 and LN18 cells. Cells were treated with FF (25 µM), irradiation (3 Gy for U87 cells, 4 Gy for LN18 cells) and a combined treatment. Mean fluorescence intensity and percentage of CD36 positively stained U87 Ai-ii) and LN18 cells Bi-ii). Mean values and standard deviation (± SD) of 4 biological replicates (n=4).


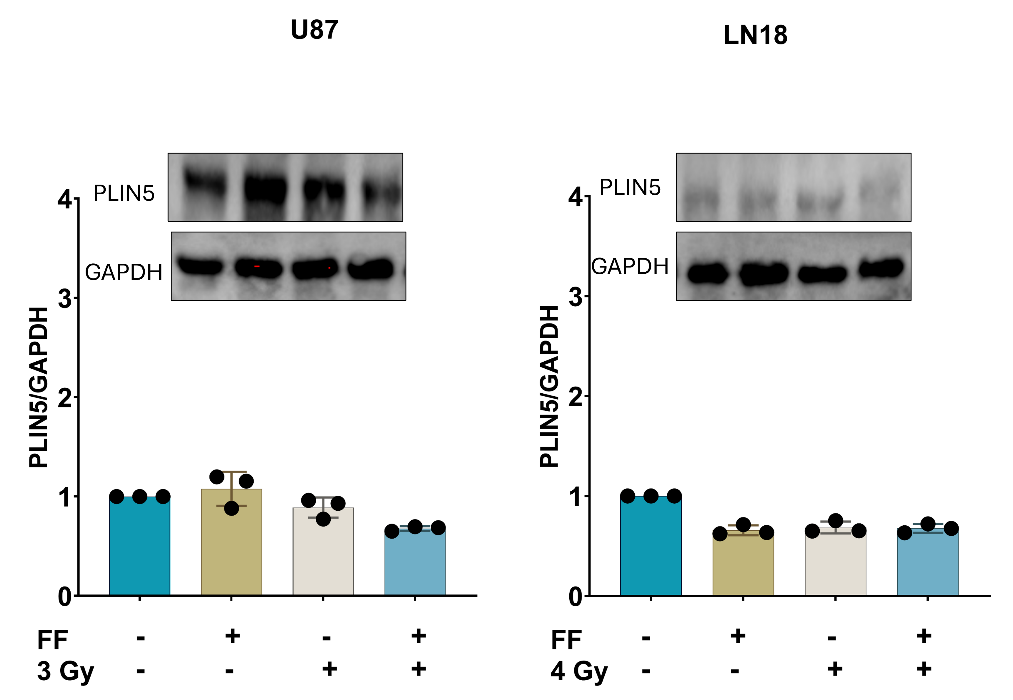


Figure S5. Effect of FF and RTx on the expression of PLIN5 in U87 (left panel) and LN18 (right panel) cells. Cells were treated with FF (25 µM), irradiation (3 Gy for U87 cells, 4 Gy for LN18 cells) or a combined treatment. GAPDH served as a loading control (lower blot).

*
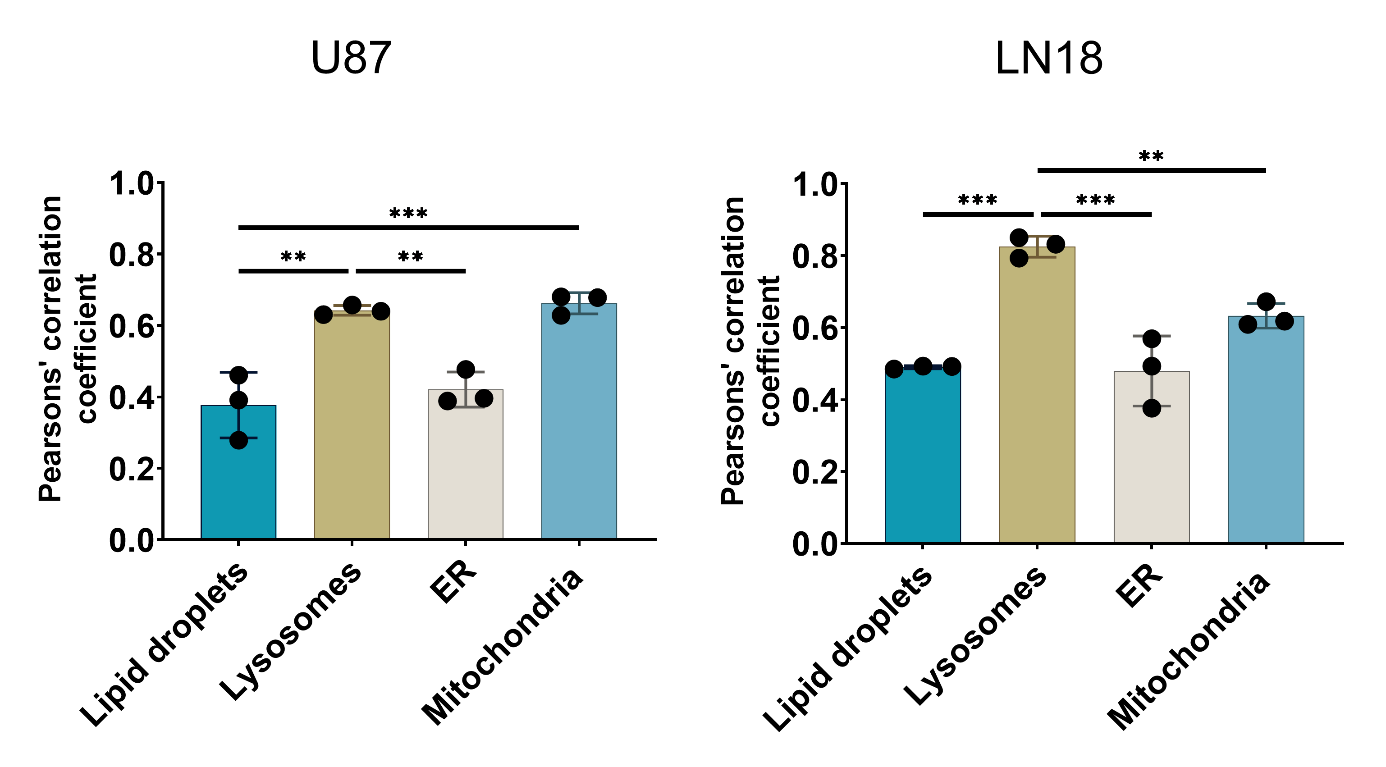
*

Figure S6. Quantitative analysis of the colocalization of FF-loaded CmEVs and subcellular organelles in U87 and LN18 cells. Analysis of Pearson’s correlation coefficient was used to estimate the degree of colocalization of FF-loaded CmEVs in lipid droplets, lysosomes, Endoplasmic reticulum (ER) and mitochondria. **p < 0.01; ***p < 0.001; ****p < 0.0001

Table S1. Antibodies, chemicals and kits used in this study. Fluorescence-activated cell sorting (FACS), Western blot (WB).

| REAGENT or RESOURCES | SOURCE | IDENTIFIER |
| --- | --- | --- |
| **Antibodies** | | |
| Actin, WB, dil1:10000 | Sigma-Aldrich | A2228 |
| Alexa Fluor® 488 AffiniPure™ Goat Anti-Rabbit IgG (H+L) intracellular dil1:10000 | Jackson ImmunoResearch | 111-545-144 |
| Anti-human cmHsp70.1, FACS | Multimmune GmbH | |
| CD36-FITC, FACS, undiluted | Miltenyi Biotec | 130-120-064 |
| CD63, FITC, FACS, dil 1:25 | Immunotech | B92467 |
| CD81, FITC, FACS, dil 0.5:15 | Immunotech | B25329 |
| CD9, FITC, FACS, undiluted | Immunotech | IM1755U |
| DGAT1, WB, dil 1:5000 | Abcam | ab181180 |
| GammaH2AX, WB, dil 1:1000 | Cell Signaling Technology | 2577 |
| GAPDH, WB, dil 1:1000 | Santa Cruze | Sc-47724 |
| FABP7, WB, dil 1:1000 | Cell Signaling Technology | 13347 |
| CD9, WB, dil 1:1000 | Cell Signaling Technology | 13174 |
| CD63 Exosome Capture Beads | Abcam | Ab239686 |
| CD9 Exosome Capture Beads | Abcam | Ab239685 |
| CD81 Exosome Capture Beads | Abcam | Ab239687 |
| GPAT4, WB, dil 1:1000, intracellular 1:2000 | Cell Signaling Technology | 66933 |
| Grp94, WB, dil 1:1000 | Cell Signaling Technology | 2104 |
| HRP-conjugated rabbit anti-mouse immuno-globulins, WB, dil:1:2000 | Dako-Agilent, Santa Clara | P0260 |
| HRP-conjugated swine anti-rabbit immu-noglobulins, WB, dil:1:1000 | Dako-Agilen | P0217 |
| Mouse IgG1 (FITC Isotype), FACS, undiluted | BD | 349041 |
| TSG101, WB, dil 1:1000 | Invitrogen | PA5-31260 |
| **Chemical reagents** | | |
| Bovine Serum Albumin (BSA) | Sigma-Aldrich | A7030 |
| Dulbecco's Modified Eagle Medium-high glucose (DMEM) | Sigma-Aldrich | D6429 |
| Aldehyde/Sulfate latex, 4% w/v 4µm | Invitrogen | A37304 |
| Dulbecco's Phosphate Buffered Saline (PBS) | Sigma-Aldrich | D8537 |
| ER-Tracker™ Red (1:1000) | Thermo Fisher Scientific | E34250 |
| Fenofibrate | Sigma-Aldrich | F6020 |
| Fetal Bovine Serum | Sigma-Aldrich | F7524 |
| Formaldehyde solution | Carl ROTH | P733.2 |
| HCS LipidTOX™ Red Neutral Lipid Stain (1:200) | Invitrogen | H34476 |
| Hochest33342 | Invitrogen | H3570 |
| LysoTracker™ Red DND-99 (1:1000) | Thermo Fisher Scientific | L7528 |
| MitoTracker™ (1:1000) | Thermo Fisher Scientific | M7514 |
| Penicillin-Streptomycin | Sigma-Aldrich | P4333 |
| Phosphatase inhibitor cocktail Tablets (PhosphoSTOP) EASYpack | Roche Diagnostic GmbH | 04906837001 |
| Pierce™ Radioimmunoprecipitation assay (RIPA buffer) | ThermoFisher Scientific | 89901 |
| PKH67 Green Fluorescent Cell Linker Kit for General Cell Membrane Labeling | Sigma-Aldrich | PKH67GL |
| Polyvinylidene fluoride membranes (PVDF) | GE healthcare life sciences | 10600021 |
| Protease inhibitor Cocktail Tablets (complete Tablets, Mini EDTA-Free, EASYpack) | Roche Diagnostic GmbH | 04693159001 |
| Restore™ PLUS Western Blot Stripping Buffer | ThermoFisher Scientific | 46430 |
| Roti-block | Carl ROTH | A151 |
| Trypsin -EDTA | Sigma-Aldrich | T4299 |
| **Kits** | | |
| Annexin V-FITC Apoptosis Staining | Abcam | ab14085 |
| DHE (Dihydroethidium) Assay Kit - Reactive Oxygen Species | Abcam | ab236206 |
| TMRE-Mitochondrial Membrane Potential Assay | Abcam | ab113852 |
| CellTiter Glo | Promega | G7571 |
| Pierce™ BCA Protein Assay Kit | Thermofisher Scientific | 23225 |
| Pierce™ ECL Western Blotting-Substrate | ThermoFisher Scientific | 32209 |
| DCFDA - Cellular ROS Assay | Abcam | ab113851 |
| DHE (Dihydroethidium) Assay Kit | Abcam | ab236206 |
